# Structural and Spectroscopic Study of Benzoperimidines Derived from 1-Aminoanthraquinone and Their Application to Bioimaging

**DOI:** 10.3390/molecules30224472

**Published:** 2025-11-19

**Authors:** Elena Kirilova, Armands Maļeckis, Muza Kirjušina, Ligita Mežaraupe, Ilze Rubeniņa, Aija Brakovska, Veronika Pavlova, Sanita Kecko, Inta Umbraško, Vladimir Kiyan, Lyudmila Lider, Aleksandrs Pučkins, Sergey Belyakov

**Affiliations:** 1 Department of Environment and Technologies, Faculty of Natural Sciences and Healthcare, Daugavpils University, LV-5401 Daugavpils, Latvia; 2 Department of Ecology, Institute of Life Sciences and Technology, Daugavpils University, LV-5401 Daugavpils, Latvia; 3 Laboratory of Biodiversity and Genetic Resources, National Center for Biotechnology, 13/5 Kurgalzhynskoye Road, Astana 010000, Kazakhstan; 4 Faculty of Veterinary Medicine and Animal Husbandry Technology, S. Seifullin Kazakh Agro Technical Research University, 62 Zhenis Avenue, Astana 010011, Kazakhstan; 5 Latvian Institute of Organic Synthesis, Aizkraukles Str. 21, LV-1006 Riga, Latvia

**Keywords:** fluorescence, anthraquinone, benzoperimidine, crystal structure, toxicology, confocal laser scanning microscopy

## Abstract

In this research, we studied the synthesis and characterization of a novel amidine derivative of benzoperimidine derived from 1-aminoanthraquinone, focusing on its emission properties and potential applications in confocal laser scanning microscopy. The synthesized compound exhibited pronounced solvatochromic behavior in various solvents. Spectroscopic analysis, including ^1^H-, ^13^C-, and mass spectrometry, confirmed the chemical structure. The structure of three compounds was also determined using X-ray diffraction analysis; this study revealed the structural features of these substances in the solid state. The compound’s antimicrobial activity was evaluated using the agar diffusion method with the bacterium *Bacillus subtilis* subsp. *Spizizenii*. Furthermore, the study introduces a dye designed for imaging of the parasitic flatworm *Opisthorchis felineus*, demonstrating its potential in visualizing biological specimens.

## 1. Introduction

Functionalized benzanthrone (7*H*-benzo[*de*]anthracen-7-one) dyes of donor-π-acceptor (D-π-A) architecture have been the subject of considerable study over the past few decades. Such donor-donating functional groups include, but are not limited to, amino [1,2], amido [3,4], amidino [5], aminophosphono [6,7], imino [8,9], alkynyl [10], alkoxy or aryloxy [11], and thioether [12] substituents. The distinctive photophysical properties—photostability, high quantum yields, large Stokes shifts, and high absorption coefficients—of these benzanthrone derivatives make them valuable for applications in sensing and dyeing. A recent review compiled and analyzed the progress of the past three decades in terms of the direct synthesis of functionalized benzanthrone compounds, modification of the benzanthrone core, their luminescent properties, and related applications [13].

**Figure 1:**
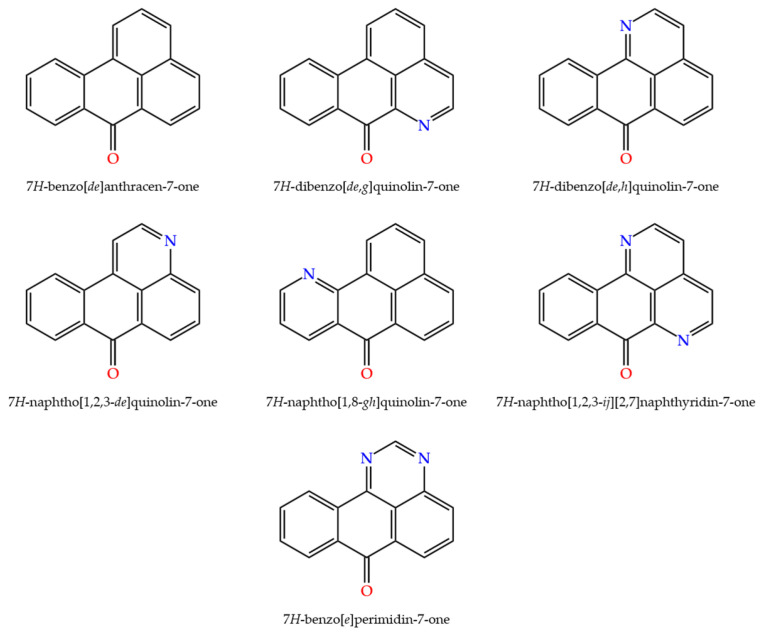
The key (di)(aza)benzanthrone derivatives.

Benzanthrone derivatives featuring nitrogen atoms in the molecular core (see Figure 1), known as azabenzanthrones, have also been documented in the literature. Such compounds include the naturally occurring 6-azabenzanthrone (7*H*-dibenzo[*de*,*g*]quinolin-7-one), also known as oxoaporphine, and 1-azabenzanthrone (7*H*-dibenzo[*de*,*h*]quinolin-7-one), also known as oxoisoaporphine, which are alkaloids with anti-inflammatory, antiprotozoal, and anticancer activities [14,15,16,17,18,19,20,21,22,23,24,25,26,27,28,29]. The synthesis of substituted 3-azabenzanthrone derivatives and theoretical studies on their optical and nonlinear optical properties have also been reported [30,31]. More recently, the synthesis of 11-azabenzanthrone and its amino derivatives as photosensitizers for cancer therapy has been described [32].

It is possible to introduce several nitrogen atoms into the benzanthrone core. For example, sampangine or 1,6-diazabenzanthrone (7*H*-naphtho [1,2,3-*ij*][2,7]naphthyridin-7-one) derivatives are structurally unique natural alkaloids with versatile biological activities, ranging from heme inhibition and reactive oxygen species (ROS) generation to promising antifungal and anticancer effects [33,34,35]. Similarly, 1,3-diazabenzanthrone (7*H*-benzo[*e*]perimidin-7-one) derivatives were studied as DNA-intercalating agents with potential anticancer activity [36]. These molecules can be synthesized from 1-aminoanthraquinone by condensation with urea or dimethylacetamide, followed by cyclization. Although the therapeutic use of aza- and diazabenzanthrone derivatives is limited by their potential toxicity and metabolic factors, ongoing research into these derivatives is paving the way for novel and more effective compounds.

While the synthesis and bioactivity profiles of aza- and diazabenzanthrones have been reported, their luminescent properties have rarely been investigated. Beyond their biological activity, azabenzanthrones also exhibit intriguing electronic and optical characteristics that distinguish them from their parent benzanthrone analogs. The introduction of one or more nitrogen atoms into the aromatic framework modulates the electron distribution and intermolecular interactions, possibly resulting in an enhanced charge-transfer character and better visualization of biological specimens in confocal laser-scanning microscopy (CLSM). This microscopy method is widely used in scientific communities to study various biological processes. In consideration of the increase in human food-borne parasitic diseases, the diagnostic and detailed investigation of these causative agents remains an important aspect of modern life sciences.

Among the functionalized benzanthrone derivatives, the amidine group was found to exhibit favorable photophysical properties and robust performance as a CLSM dye [37]. To develop new luminophores with improved properties for practical use in CLSM, we synthesized a 7*H*-benzo[*e*]perimidin-7-one cyclic amidine derivative and investigated its photophysical properties.

## 2. Results and Discussion

### 2.1. Synthesis

Since the last century, 1-aminoanthraquinone has been of great practical importance, due to the possibility of its functionalization and the creation of new substances, including heterocyclic compounds [38]. Among the numerous heterocyclic derivatives, perimidines represent an interesting class of N-heterocycles that have seen significant development in recent years, due to their wide application in the life sciences, medicine, and industrial chemistry [39,40]. Various new approaches to the synthesis of benzoperimidines and their derivatives have been developed from 1-aminoanthraquinone [36,41] or 1-iodoanthraquinone [42].

In this work, we used a previously developed method based on the cyclization of an amidine obtained from 1-aminoanthraquinone with subsequent amination of the resulting benzoperimidine.

**Scheme 1:**
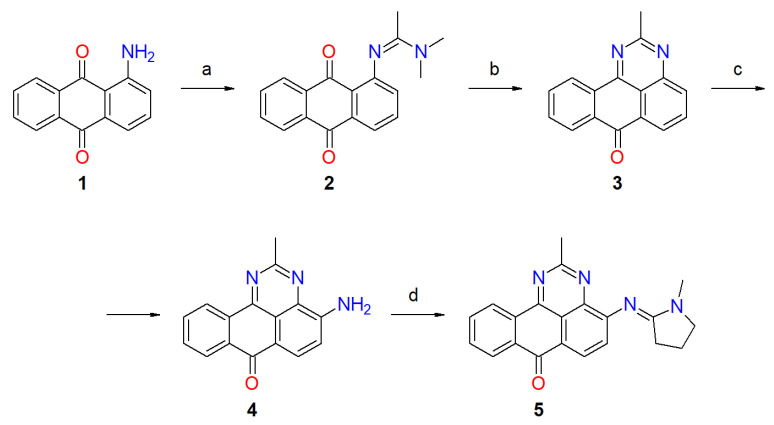
Synthesis of target benzoperimidine derivative **5**. The reaction conditions and yields: (a): MeCN, DMAC, POCl_3_, 95%; (b): NH_4_OAc, EtOH, 90%; (c): NH_2_OH, diglycol, 73%; (d): *N*-methylpyrrolidone, POCl_3_, 63%.

The synthesis of target compound **5** (Scheme 1) was guided by prior studies [41,43]. Commercially available 1-aminoanthraquinone (**1**) was first transformed into intermediate **2** through a Vilsmeier–Haack-type reaction with phosphorus oxychloride in the presence of *N,N*-dimethylacetamide. Subsequent condensation of **2** with ammonium chloride in methanol yielded intermediate **3**, which was then directly aminated using hydroxylamine in diethylene glycol. The resulting amino derivative opens up broad and previously underexplored possibilities for modification via alkylation, acylation, condensation reactions, etc. Finally, applying the same reaction conditions used for the formation of **2**, intermediate **4** was converted into the desired compound **5** via reaction with *N*-methylpyrrolidone. The chemical structures of the synthesized compounds were confirmed using NMR and IR spectroscopy methods, as well as mass spectrometry.

For the amino group (NH_2_), the obtained infrared spectra showed two stretching vibration bands around 3300–3420 cm^−1^ for amine **4**, two stretching vibration peaks of anthraquinone carbonyl groups (C=O) for derivatives **2**–**3** at around 1630–1670 cm^−1^, and one for benzoperimidine derivatives **4** and **5** carbonyl group at around 1660–1670 cm^−1^.

Confirmation of the obtained compounds’ structures was achieved using ^1^H-NMR spectroscopy. The characteristic signals of aromatic protons were identified. Hydrogens within the methyl groups of compound **5** appeared as two singlet signals at 3.20 and 3.02 ppm. The protons of the pyrrolidine ring showed two triplet signals at 3.55 and 2.53 ppm and a quintet at 2.06 ppm.

The mass of the synthesized compounds, determined from the results obtained from high-resolution mass spectrometry, was verified to match the calculated values.

### 2.2. X-Ray Crystallographic Study

**Figure 2:**
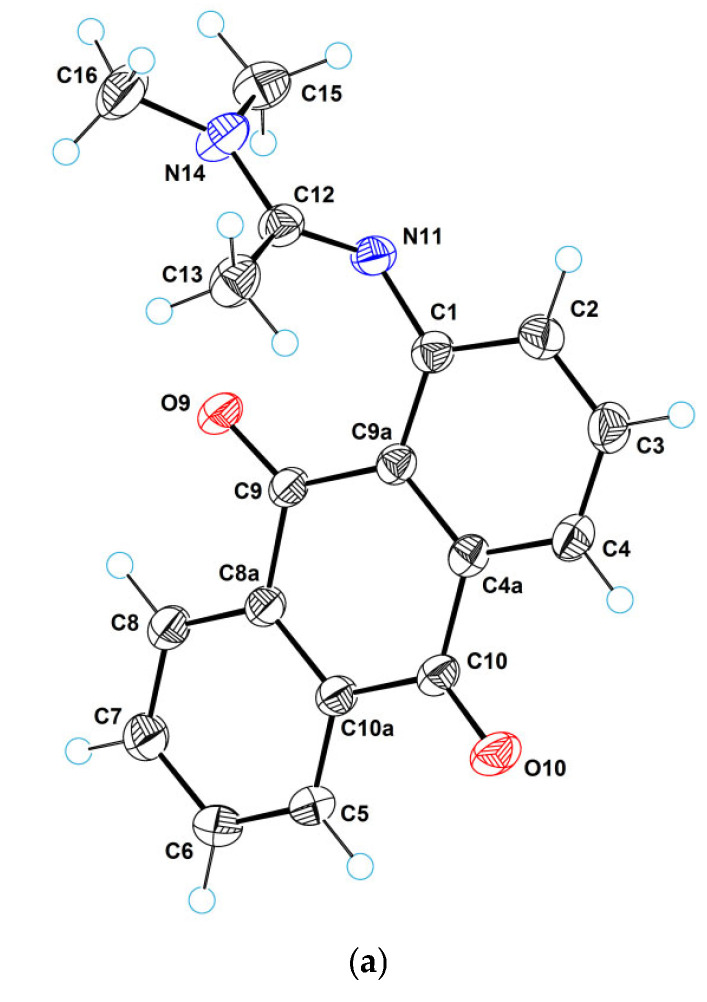

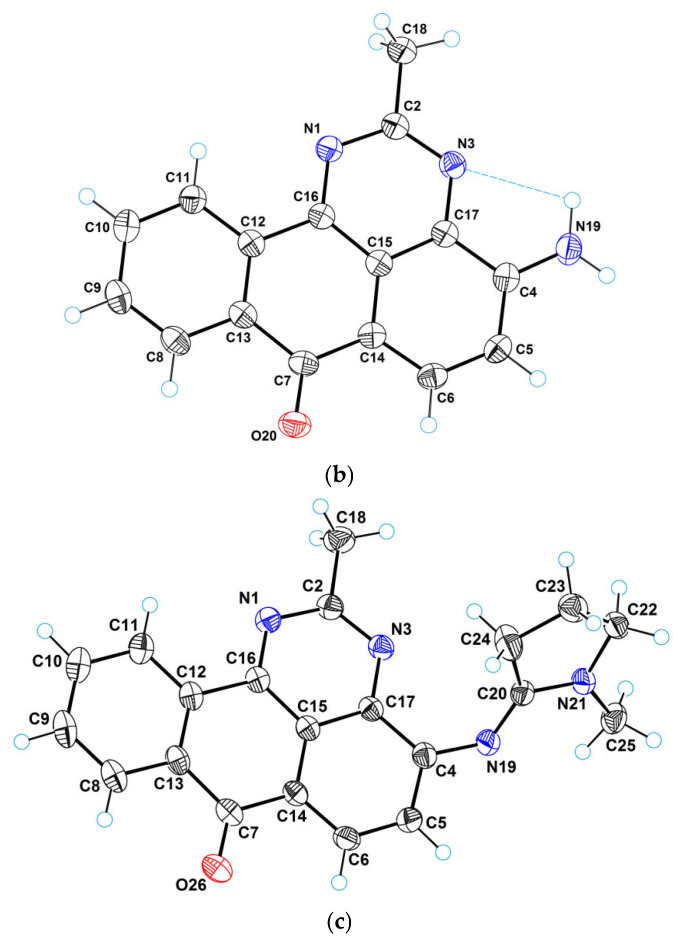
ORTEP diagrams for molecules **2** (**a**), **4** (**b**), and **5** (**c**).

Figure 2 provides a perspective view of molecular structures **2**, **4**, and **5**, respectively, with thermal ellipsoids and the atom numbering scheme. Molecule **2** is characterized by the usual geometry of the anthraquinone fragment. The planar dimethylacetimidamide group cannot be coplanar with the anthraquinone system, due to steric hindrance. That is why this group is turned and forms a dihedral angle of 81.9(2)° with the anthraquinone system. Such a high value of the dihedral angle does not promote conjugation; therefore, the length of the C1−N11 bond (1.384(1) Å) exceeds the standard value for Car−Nsp^2^ bonds [44].

**Figure 3:**
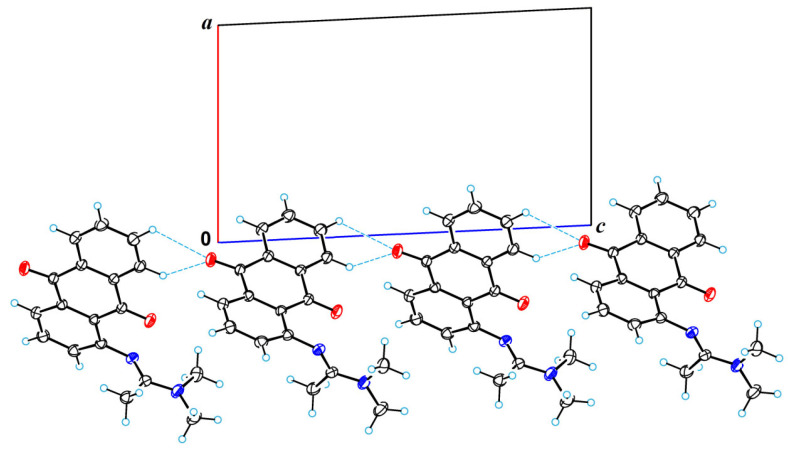
A molecular chain in the crystal structure **2** viewed along the monoclinic axis.

In the crystal structure of compound **2**, intermolecular bifurcated hydrogen bonds of CH···O···HC type are observed. These bonds are moderate, with the following parameters: C7···O10 = 3.181(1) Å (H7···O10 = 2.59(1)Å, C7−H7···O10 = 119(1)°); C8···O10 = 3.104(2) Å (H8···O10 = 2.43(2) Å, C8−H8···O10 = 125(2)°). By means of these bonds, molecular chains are formed in the crystal structure along the longest lattice parameter (along the parameter c). These chains are shown in Figure 3.

**Figure 4:**
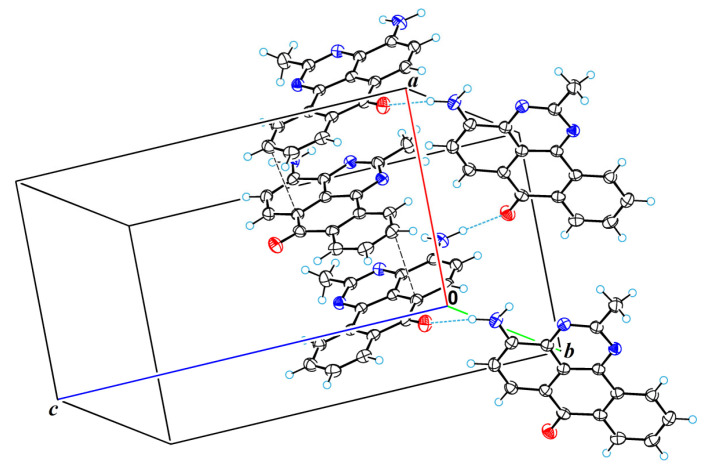
A fragment of molecular packing in the crystal structure **4** showing the intermolecular H-bonds and the shortest atom–atomic contacts.

The polycyclic aromatic system in derivative **4** is planar and resembles the polycyclic system in benzanthrone. The atoms C18, N19, and O20 lie in the plane of the polycyclic system. The amino group is characterized by trigonal planar geometry and participates in the formation of hydrogen bonds. One of these bonds (the bond N19H···N3) is intramolecular with the length of 2.741(2) Å (H19b···N3 = 2.36(2) Å, N19−H···N3 = 104(3)°). The second hydrogen bond is intermolecular NH···O type bond with the length of 2.918(2) Å (H19a···O20 = 1.98(2) Å, N19−H19a···O20 = 164(2)°). Due to this intermolecular bond, molecular chains are formed in the crystal structure along the smallest lattice parameter (along parameter a). Intermolecular π-π stacking interactions with the smallest atom-atomic contact of 3.311(3) Å (C10···C14 contact) are also observed in the crystal structure. Figure 4 shows a fragment of the packing of **4** molecules in the crystal lattice.

### 2.3. Photophysical Properties

**Figure 5:**
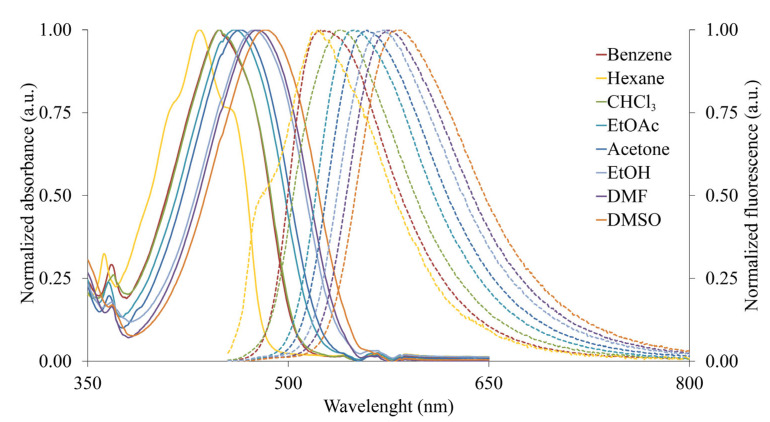
The UV–Vis absorption and fluorescence emission (dotted lines) spectra of compound **4** in various organic solvents.

**Figure 6:**
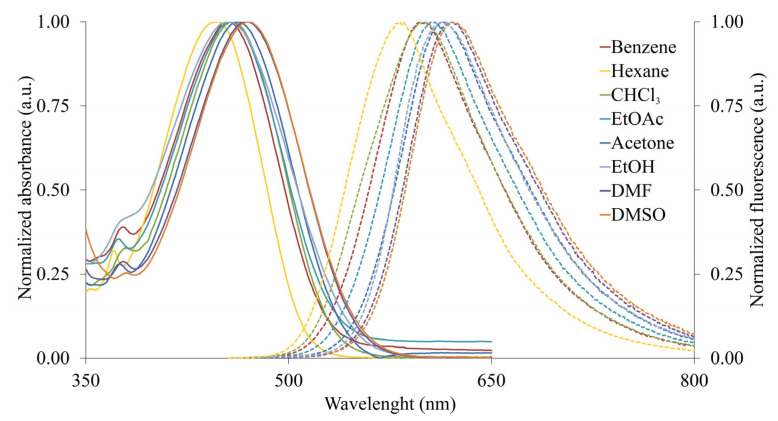
The UV–Vis absorption and fluorescence emission (dotted lines) spectra of compound **5** in various organic solvents.

To investigate the photophysical characteristics of the synthesized compounds, UV–Vis absorption and fluorescence emission spectra in solvents of varying polarity (benzene, chloroform, ethyl acetate, acetone, *N,N*-dimethylformamide, dimethyl sulfoxide, and ethanol) were acquired. Table 1 provides a summary of the data, including the absorption maxima, molar attenuation coefficients, and emission maxima. For the studied compounds **4** and **5**, the absorption and fluorescence spectra are shown in Figure 5 and Figure 6.

The intermediates amidine **2** and perimidine **3** exhibit absorption in the region of 400–417 nm and 447–480 nm, respectively, and are practically non-luminescent. The introduction of an amino group into the benzoperimidine system results in the appearance of fluorescence in product **4** in the range of 520–585 nm. Furthermore, the synthesized cyclic amidine **5** also exhibits an emission that is bathochromically shifted by 60–70 nm compared to the initial amine 4. The largest Stokes shift of 5691 cm^−1^ is observed for derivative **5** in ethanol, and for amine **4**, it is 3598 cm^−1^. The synthesized amidine **5** exhibits more pronounced emission and solvatochromic effects, which can be explained by more efficient intramolecular charge transfer from the amidine residue to the carbonyl group, compared to the transfer in the molecule of the starting amine **4**. Thus, the substitution of hydrogen atoms contributes to the formation of more emissive and polarity-sensitive derivatives of 4-aminoperimidine.

### 2.4. Antimicrobial Activity

In recent years, particular attention has been paid to the use of anthrone derivatives in some biological studies, which has led to the need to evaluate their biological activity. Thus, the microbiological activity of some ammonium salts and metal complexes of anthraquinone and benzanthrone has been studied with the aim of developing substances for the production of colored antibacterial dressings and other antibacterial textile products [45,46,47,48]. The antimicrobial activity of target compounds is often studied in vitro using the agar diffusion method with various bacterial and yeast cultures [49].

**Figure 7:**
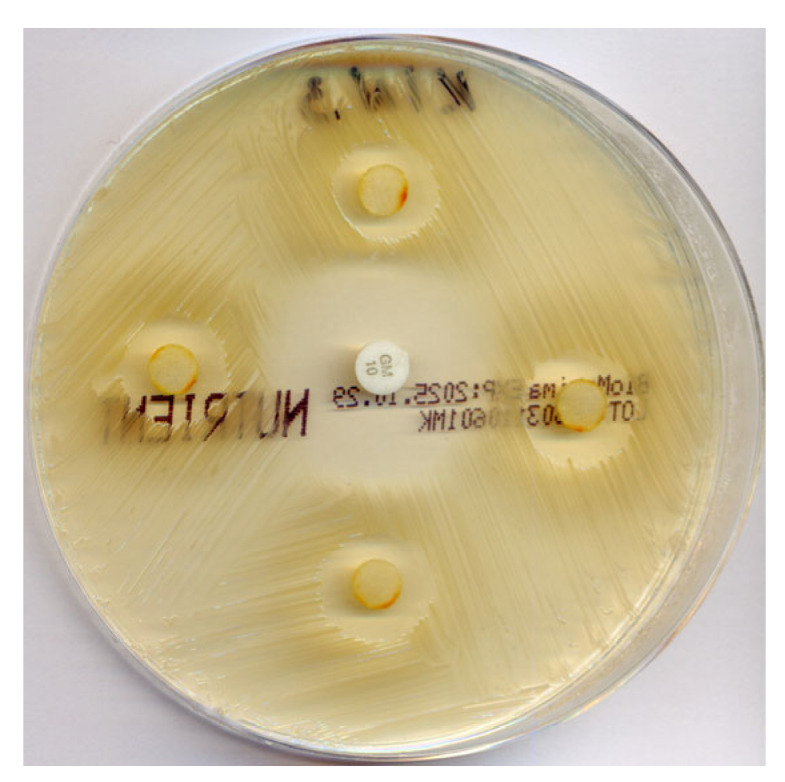
Zones of inhibition of the growth of *Bacillus subtilis* subsp. *Spizizenii* with 10 µg of compound **5**.

In this study, the antimicrobial activity of the obtained compounds was assessed using the Gram-positive bacteria *Bacillus subtilis* subsp. *Spizizenii*. For the growth of *Bacillus subtilis* subsp. S*pizizenii* with gentamicin (Gm, 10 µg), the inhibition zone diameters were 18–26 mm. As for the growth of *Bacillus subtilis* subsp. spizizenii with compounds **1**, **2**, **3**, and **4** (10 µg), the inhibition zones were not detected. In turn, inhibition zone diameters with compound **5** (10 µg) ranged from 12 to 14 mm. Antimicrobial tests indicated the good antimicrobial activity of the synthesized compound **5**, where the inhibition zones of the bacteria *Bacillus subtilis* subsp. *Spizizenii* were approximately half as small as those of gentamicin (see Table 2 and Figure 7).

Thus, antimicrobial tests showed that 1-aminoanthraquinone does not exhibit antimicrobial activity, in contrast to its substituted amide, the activity of which against *B. subtilis*, as previously established [45], is only slightly inferior to that of the standard (gentamicin). It is obvious that an increase in the conjugated aromatic system leads to an increase in antimicrobial activity. Therefore, the antimicrobial activity of substituted diazabenzanthrone **5** is similar to the activity observed for benzanthrone derivatives [46].

### 2.5. Imaging of Opisthorchis felineus

According to the most recent estimates from the Global Burden of Disease Study, the total number of food-borne trematode infections, including opisthorchiasis, has reached approximately 44.5 million cases [50]. Among members of the family Opisthorchiidae, the most medically important species for humans are *Opisthorchis felineus*, *O. viverrini,* and *Clonorchis sinensis* [51]. Recent reviews suggest that about 1.6 million people are infected with *O. felineus*, 9–10 million with *O. viverrini*, and about 15 million with *C. sinensis* [52].

Diagnosis of opisthorchiasis in humans traditionally relies on the microscopic detection of parasite eggs in feces; however, its sensitivity decreases in light or chronic infections. Modern serological tests (ELISA) and antigen-detection assays demonstrate markedly higher diagnostic accuracy, with reported sensitivities of 94–100% and specificities of up to 99% [53,54,55]. Moreover, new urine antigen rapid diagnostic tests (OV-RDT) have shown comparable performance and can facilitate field-level diagnosis in endemic areas [55]. The implementation of such sensitive and affordable tools is critical for the early detection of asymptomatic and chronic infections.

Chronic opisthorchiasis is associated with biliary tract pathologies, including cholangitis, fibrosis, and cholangiocarcinoma (CCA) [56,57]. Experimental studies further demonstrate that *O. felineus* can induce biliary intraepithelial neoplasia, a precancerous lesion of the bile ducts, and produce oxysterol-like metabolites capable of causing DNA damage in host tissues [58].

*O. felineus* is regarded as a potential carcinogenic agent comparable to *O. viverrini* and *C*. *sinensis* [59]. Thus, given the high prevalence of opisthorchiasis and its association with cholangiocarcinoma, *O. felinius* was selected as the model organism for the development of a sensitive diagnostic tool using CLS microscopy.

**Figure 8:**
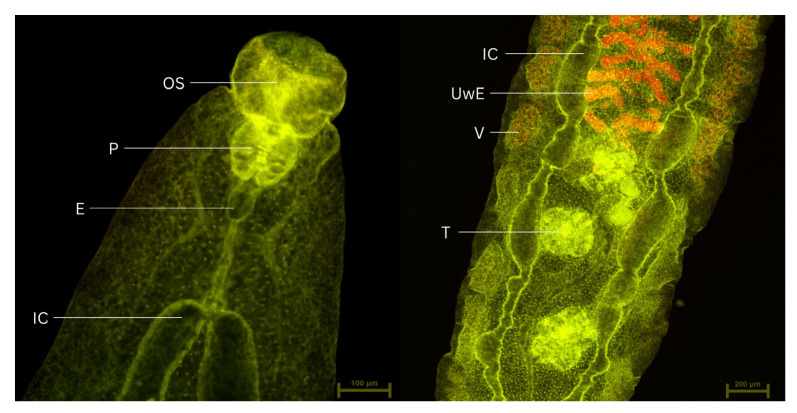
Adult *Opisthorchis felineus* stained with the examined dye **5**, showing the internal structure: OS: oral sucker, P: pharynx, E: esophagus, IC: intestinal caeca, VG: vitellaria glands, O: ovary, UwE: uterus filled with eggs, T: testis. CLSM was performed using 488 nm and 638 nm lasers.

The developed fluorophore **5** produced high-quality and high-resolution images, emphasizing the key internal structures of *O. felineus* (see Figure 8). Dye **5** effectively stained the internal organs, including the digestive and reproductive systems, and allowed clear visualization of the muscular structures at magnifications up to ×600.

The results revealed elongated, flattened, and leaf-like trematodes with tapered anterior ends, which were typical of *O. felineus*. The specimen exhibited two small circular suckers—an oral sucker positioned almost terminally at the anterior end and a ventral sucker (acetabulum) lying slightly behind the anterior third part, which is also described as a key diagnostic feature separating *Opisthorchis* from *Clonorchis*.

Within the digestive system, the following were visualized: a short prepharynx, a globular pharynx behind the oral sucker, a short esophagus, and bifurcated thin straight intestinal caeca posteriorly nearly to the tail end, exhibiting the highest fluorescence intensity with strong red emission under 638 nm excitation. Within these regions, the fluorophore’s high tissue penetration and strong affinity for peptide- and protein-rich regions were shown.

The reproductive system was well obtained. The ovary was visualized as small, oval, and located in the midline just anterior to the testes. Two large, spherical, and symmetrical testes were located posteriorly. The uterus was coiled, filled with numerous operculated eggs occupying the central region anterior to the testes, in general situated between the ventral sucker and the testes. The vitellaria were lateral, extending from the midbody to the posterior end, providing yolk for the eggs. The eggs were small ovals, operculated with a prominent opercular shoulder at one end and a small terminal knob at the opposite end.

The body tegument was smooth and translucent, sometimes with faint granulation visible under higher magnification.

However, spikes in the muscle system were not clearly observed. Overall, the fluorophore enabled detailed visualization of the preserved specimens, yielding superior resolution and tissue contrast, particularly for muscular and reproductive structures.

## 3. Materials and Methods

### 3.1. Materials and Measurements

All of the reagents and solvents were obtained commercially and used without any additional purification. The assessment of the progress of reactions and the purity of the synthesized compounds was performed using TLC on MERCK (Boston, MA, USA) Silica gel 60 F254 plates in hexane/acetone (1:2) as an eluent and visualized under UV light. Melting points were determined using a METTLER TOLEDO™ (Greifensee, Switzerland) Melting Point System MP70 apparatus.

The IR spectrum was recorded using a Thermo Scientific Nicolet iS50 Spectrometer (Waltham, MA, USA, ATR accessory; no. of scans: 64; resolution: 4; data spacing: 0.482 cm^−1^). ^1^H- and ^13^C- NMR spectra were recorded using a Bruker Avance 500 MHz (Bruker Corporation, Billerica, MA, USA) in CDCl_3_ at ambient temperature, with solvent peaks as the internal reference. The chemical shift (δ) values are reported in ppm. High-resolution accurate mass measurements were performed employing an Orbitrap Exploris 120 (Thermo Fischer Scientific) operating at Full Scan mode at 120,000 resolution.

The fluorescence emission spectra were recorded using an FLSP920 (Edinburgh Instruments Ltd., Livingston, UK) spectrofluorometer in the visible range 450–800 nm, and the absorption spectra were obtained using the UV–visible spectrophotometer SPECORD^®^ 80 (Analytik Jena AG, Jega, Germany). The spectral properties of the investigated compound were measured at an ambient temperature in quartz cuvettes of 10 mm in hexane, benzene, chloroform, ethyl acetate (EtOAc), acetone, ethanol (EtOH), dimethyl sulfoxide (DMSO), and dimethylformamide (DMF) with a concentration of 10^−5^ M. The quantum yields were measured relative to rhodamine 101 as the standard.

### 3.2. Synthesis and Characterization

*2-Methyl-4-amino-7H-benzo[e]perimidin-7-one* (**4**) was obtained by condensation of 1-aminoanthraquinone (**1**) with *N,N*-dimethylacetamide and subsequent cyclization of the resulting (*N*’-(9,10-dioxo-9,10-dihydroanthracen-1-yl)-*N,N*-dimethylethanimideamide (**2**) to 2-methyl-7H-benzo[e]perimidin-7-one (**3**) and its amination, according to the procedure described by Yan-Ping Li and coworkers [41].

*2-Methyl-4-((1-methylpyrrolidin-2-ylidene)amino)-7H-benzo[e]perimidin-7-one* (**5**) was synthesized as follows. POCl_3_ (1.86 mL, 20 mM) was added dropwise to a stirring solution of *N*-methylpyrrolidone (2.41 mL, 25 mM) in 10 mL of acetonitrile in a 50 mL round-bottom flask and left to stir for an hour. 2-methyl-4-amino-*7*H-benzo[*e*]perimidin-7-one (2.61 g, 10 mM) and 10 mL of acetonitrile were then added to the solution in one portion. The mixture was then heated at 50 °C for 2 h, cooled, poured onto 100 mL of water, and basified with 10% NaOH. The precipitate formed was filtered off, washed with water, and dried in air. Recrystallization from xylenes afforded a red solid in 63% yield, with a melting point of 179 °C and R*f* = 0.57 (TLC, silica gel, DCM/acetone 1:1). ^1^H NMR (500 MHz, Chloroform-*d*) δ 8.96 (d, *J* = 7.7 Hz, 1H), 8.49 (d, *J* = 8.1 Hz, 1H), 8.46 (d, *J* = 7.7 Hz, 1H), 7.81 (t, *J* = 7.1 Hz, 1H), 7.75 (t, *J* = 7.4 Hz, 1H), 7.36 (d, *J* = 8.1 Hz, 1H), 3.55 (t, *J* = 6.9 Hz, 2H), 3.20 (s, 3H), 3.02 (s, 3H), 2.53 (t, *J* = 7.8 Hz, 2H), 2.06 (p, *J* = 7.4 Hz, 2H). ^13^C NMR (APT, 126 MHz, Chloroform-*d*) δ 181.43 (C=O), 163.86 (C), 156.89 (C), 156.35 (C), 144.30 (C), 135.01 (C), 134.41 (C), 133.15 (CH), 131.96 (CH), 130.41 (CH), 127.54 (CH), 125.42 (CH), 124.65 (CH), 121.60 (C), 119.12 (C), 51.71 (CH_2_), 31.88 (CH_3_), 29.09 (CH_2_), 27.18 (CH_3_), 19.96 (CH_2_). GC-MS: calculated for [C_21_H_18_N_4_O]: 342.2, found: 342.1.

### 3.3. Antimicrobial Activity Test

The antimicrobial activity of the compounds was tested in vitro via the agar diffusion method using the Gram-positive bacterium *Bacillus subtilis* subsp. *Spizizeni*i [49].

Nutrient agar plates were seeded with cell suspensions of the test *Bacillus Subtilis* subsp. *Spizizenii* cultures. Commercial disks with gentamicin (Gm, 10 µg) and the studied compounds 2–5 (10 µg) were used as a standard antibacterial and antifungal agent, respectively. The plates were incubated at an appropriate temperature (+30 °C) and monitored for growth for 24–48 h. The growth of microorganisms was inhibited by diffusion of the test solutions, and the antimicrobial activity was indicated by the presence of clear inhibition zones around the wells.

### 3.4. Confocal Laser Scanning Microscopy Imaging

We visualized the internal structure of 30 *Opisthorchis felineus* specimens fixed in 70% ethanol more than 5 years ago and subsequently stained with the synthesized fluorescent dye **5**.

A simple and rapid staining protocol was developed based on previous experience with other luminophores applied to biological specimens [37,60,61,62,63].

The staining procedure consisted of 4 main steps: staining, rinsing, clearing, and mounting. In the first step, each specimen was transferred from a microtube to a watch glass, and all residual fixative was removed. Subsequently, 2 mL of compound **5** fluorescent dye dissolved in ethanol was added for 5 min. In the second step, the staining solution was removed, and the specimen was rinsed 3 times with 2 mL of 70% ethanol. In the third step, after rising, the ethanol was removed, and 2 mL of the clearing agent (a 1:1 mixture of 70% ethanol and 100% xylene) was added. The clearing agent was applied for 30 s to increase the tissue transparency and improve the structural visibility. In the last step, the specimen was transferred to a microscopy slide, the clearing agent was removed, and the sample was mounted in Canada balsam. A coverslip was carefully placed over the mounted specimen.

Fluorescence imaging was performed using a confocal laser scanning microscope (Eclipse Ti-E; Nikon, Tokyo, Japan) equipped with a digital sight DS-U3 camera and an A1R MP high-speed multiphoton system (Nikon, Japan). Image acquisition and analysis were conducted using NIS-Elements AR software (version 3.2, 64-bit; Nikon, Japan). Excitation was provided by 488 nm and 638 nm lasers with FITC (500–550 nm) and Cy5 (662–737 nm) emission filters, respectively. Fluorescence signals were detected using the internal spectral detector, with the detection range set from 20 nm above the excitation wavelength to the red edge of the visible spectrum. No passive cut-off filters were applied in the optical path. Z-stack images were collected at 2.0 µm intervals, and morphological measurements were performed using the same software.

### 3.5. Single Crystal X-Ray Diffraction Analysis

For compounds **2**, **4**, and **5**, diffraction data were collected at low temperature on a Rigaku (Tokyo, Japan) XtaLAB Synergy Dualflex HyPix diffractometer using copper monochromated Cu-Kα radiation (λ = 1.54184 Å). The crystal structures were solved using direct methods [64] and refined using full-matrix least squares [65]. All non-hydrogen atoms were refined in an anisotropic approximation. For further details, see the crystallographic data for **2**, **4**, and **5** deposited at the Cambridge Crystallographic Data Center as Supplementary Publications Numbers CCDC 2470312 (for **2**), CCDC 2470313 (for **4**), and CCDC 2470314 (for **5**). These data are provided free of charge by the joint Cambridge Crystallographic Data Center and Fachinformationszentrum Karlsruhe Access Structures service.

## 4. Conclusions

In conclusion, the newly synthesized benzoperimidine derivative exhibits notable fluorescence properties with solvatochromic behavior, making it suitable for a wide variety of applications. The spectroscopic analysis confirmed the chemical structures, and the compound’s emission properties were thoroughly explored in various organic solvents. The X-ray crystallographic study of the studied compound contributes valuable information about the compound’s molecular arrangement and intermolecular interactions.

An assessment of antimicrobial activity on the soil bacterium *Bacillus subtilis* subsp. *Spizizenii* revealed a significant effect of the obtained dye on the growth of these bacteria. Overall, the synthesized compounds demonstrate potential for versatile applications, particularly in the investigation of parasitic organisms. The developed dye demonstrates strong fluorescence, high photostability, and specific affinity toward muscular and reproductive tissues, enabling clear imaging of multiple organ systems. The optimized staining and clearing protocol developed in this study ensures efficient sample preparation for CLSM and can serve as a basis for further bioimaging research using anthrone-based dyes.

## Figures and Tables

**Table 1:** Absorption maxima (extinction coefficient) and fluorescence maxima (quantum yield) of the obtained compounds **2**–**5** in organic solvents (concentration 10^–5^ M).

Solvent	2	3	4		5	
	λ_abs_, nm (lgε)	λ_abs_, nm (lgε)	λ_abs_, nm (lgε)	λ_em_ (Q), nm	λ_abs_, nm (lgε)	λ_em_ (Q), nm
Hexane	410 (3.60)	447 (3.96)	434 (4.33)	522 (0.001)	448 (4.26)	582 (0.47)
Benzene	412 (3.61)	461 (3.95)	448 (4.32)	527 (0.05)	455 (4.34)	599 (0.43)
Chloroform	411 (3.49)	489 (3.76)	449 (4.32)	540 (0.01)	458 (4.35)	600 (0.54)
EtOAc	412 (3.55)	469 (3.91)	460 (4.33)	550 (0.08)	457 (4.32)	607 (0.48)
Acetone	412 (3.52)	469 (3.91)	464 (4.37)	559 (0.06)	462 (4.30)	615 (0.20)
DMF	417 (3.38)	480 (3.94)	477 (4.35)	575 (0.04)	469 (4.28)	620 (0.16)
DMSO	417 (3.31)	484 (3.88)	483 (4.37)	583 (0.04)	471 (4.42)	622 (0.12)
EtOH	400 (3.61)	477 (3.98)	473 (4.35)	570 (0.015)	455 (4.23)	614 (0.07)

**Table 2:** Zones of inhibition of the growth of the test culture with the investigated compounds **1**–**4**.

Compound	Zones, mm/diameter
Gentamicin	22 ± 4
Compound **1**	0
Compound **2**	0
Compound **3**	0
Compound **4**	0
Compound **5**	13 ± 1

## Data Availability

Data are contained within the article or Supplementary Materials.

## Supplementary Materials

The following supporting information can be downloaded at https://www.mdpi.com/article/10.3390/molecules30224472/s1. ^1^H, ^13^C NMR spectra; MS (ESI) data.
