# Supplementary material for: Structural and Spectroscopic Study of Benzoperimidines Derived from 1-Aminoanthraquinone and Their Application to Bioimaging
Source: Molecules. 2025 Nov 19;30(22):4472. doi: 10.3390/molecules30224472 (PMC12655274; doi:10.3390/molecules30224472)
Supplement: Supplementary file 1 [file molecules-30-04472-s001.zip › molecules-3957693-supplementary.pdf]

Supplementary

Structural and Spectroscopic Study of Benzoperimidines Derived from 1-Aminoanthraquinone and Their Application to Bioimaging

Elena Kirilova [1,*], Armands Maļeckis [1], Muza Kirjušina [2], Ligita Mežaraupe [2], Ilze Rubeniņa [2], Aija Brakovska [2], Veronika Pavlova [2], Sanita Kecko [2], Inta Umbraško [2], Vladimir Kiyan [3], Lyudmila Lider [4], Aleksandrs Pučkins [1] and Sergey Belyakov [5,*]

1 Department of Environment and Technologies, Faculty of Natural Sciences and Healthcare, Daugavpils University, LV-5401 Daugavpils, Latvia
2 Department of Ecology, Institute of Life Sciences and Technology, Daugavpils University, LV-5401 Daugavpils, Latvia
3 Laboratory of Biodiversity and Genetic Resources, National Center for Biotechnology, 13/5 Kurgalzhynskoye Road, Astana 010000, Kazakhstan
4 Faculty of Veterinary Medicine and Animal Husbandry Technology, S. Seifullin Kazakh Agro Technical Research University, 62 Zhenis Avenue, Astana 010011, Kazakhstan
5 Latvian Institute of Organic Synthesis, Aizkraukles Str. 21, LV-1006 Riga, Latvia
* Correspondence: jelena.kirilova@du.lv (H.K.); serg@osi.lv (S.B.)

SYNTHESIS OF COMPOUNDS **2-4** [36]

***N'*-(9,10-Dioxo-9,10-dihydroanthracen-1-yl)-*N,N*-dimethylethanimidamide (2)**

N,N-Dimethylacetamide (2.18 g, 25 mM) was firstly dissolved in acetonitrile and phosphorus oxychloride (1.86 mL, 20 mM) was slowly added in droplets to it. After stirring for 1 h, 1-amino anthraquinone (2.23 g, 10 mM) was added. The mixture reacted for 1 h in RT and then for 6 h in 50°C under stirring. Then the liquid was transferred into ice water. The mixture was neutralized with 10% NaOH. Filtration and washing afforded compound **2** (2.74 g, 95%) as a red powder. m. p. 158-161 °C.

2-Methyl-7*H*-benzo[*e*]perimidin-7-one (3)

A solution of compound **2** (2.20 g, 7.5 mM) and ammonium acetate (2.89 g, 37.5 mM) in ethanol (40 mL) was refluxed for 2 h under stirring. Then it was cooled to room temperature, filtrated and washed with ethanol and water successively. 2-Methyl-7H-benzo[e] perimidin-7-one **3** (1.67 g, 90%) was obtained as a yellow solid with m. p. 211-212 °C.

4-Amino-2-methyl-7*H*-benzo[*e*]perimidin-7-one (4)

To a solution of compound **3** (0.5 g, 2.03 mM) and hydroxylamine hydrochloride (2.7 g, 38.86 mM) in diethylene glycol (15 mL), saturated NaOH solution was added in droplets into the mixture and then stirred at 100 °C for 15 min. After the solution was cooled to RT, the reaction mixture was poured into water. The solid was then filtrated and washed with

water. Compound **4** (0.39 g, 73%) was obtained by silica gel column chromatography as a red solid.

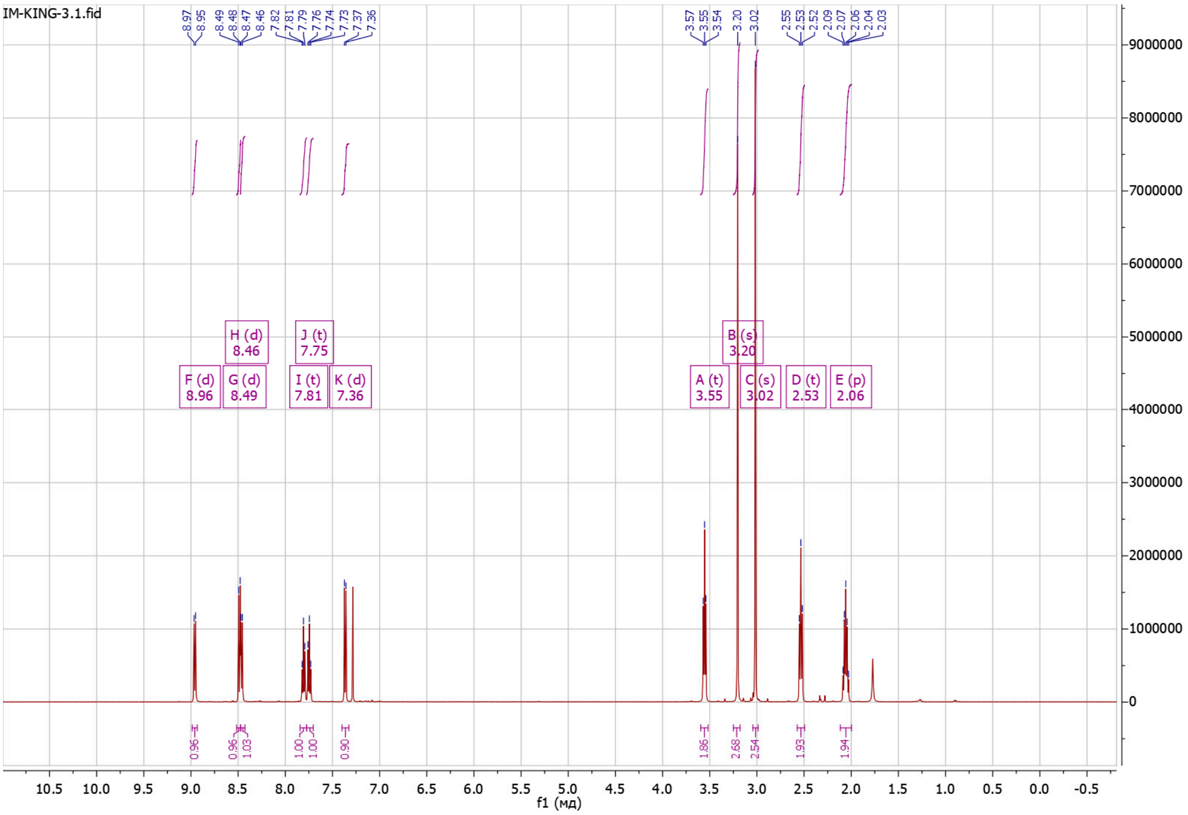

Figure S1. ^{1}H NMR (500 MHz, chloroform-*d*) spectrum of compound **5**.

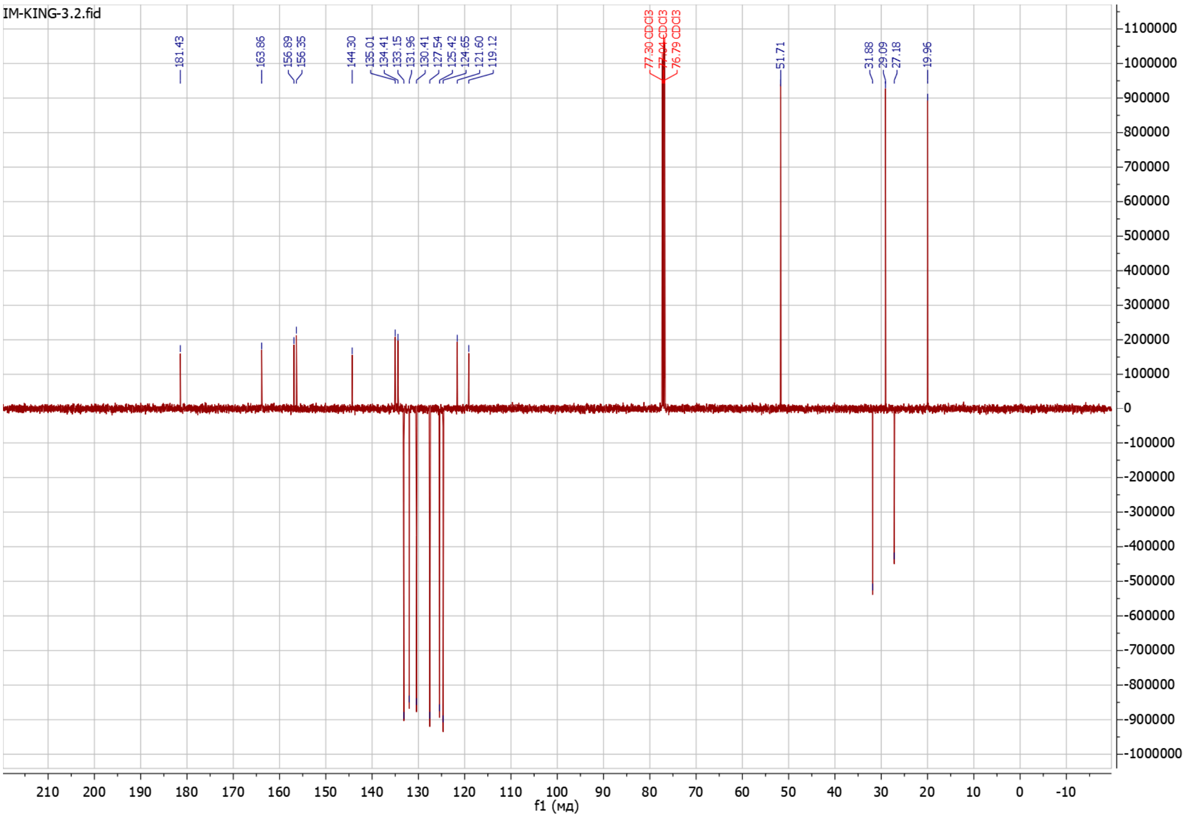

Figure S2. ^{13}C NMR (126 MHz, chloroform-*d*) spectrum of compound **5**.

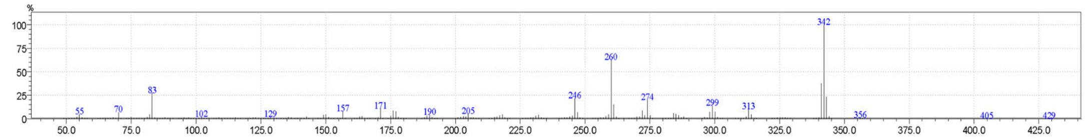

Figure S3. The mass spectrum of compound **5**.

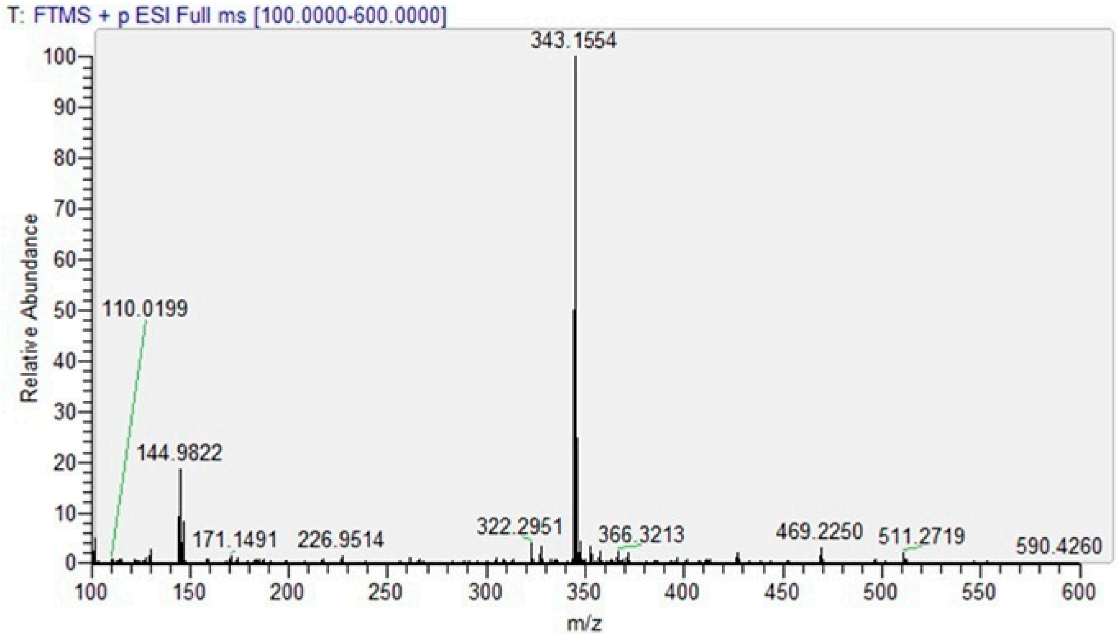

Figure S4. High-resolution mass spectrum of compound **5**.
